# Supplementary material for: NAD(P)H Quinone Oxidoreductase-1 Expression Promotes Self-Renewal and Therapeutic Resistance in Non-Small Cell Lung Cancer
Source: Genes (Basel). 2023 Feb 28;14(3):607. doi: 10.3390/genes14030607 (PMC10047941; doi:10.3390/genes14030607)

NAD(P)H Quinone Oxidoreductase-1 expression promotes self-renewal and therapeutic resistance in non-small cell lung cancer

Brian Madajewski*[1,2], Michael A. Boatman[2], Ivan Martinez[2], [3]Julia H. Carter, and Erik A. Bey*[2,3]

[1]Memorial Sloan Kettering-Cornell Center for Translation of Cancer Nanomedicine, Memorial Sloan Kettering Cancer Center, 415 East 68th Street, New York, NY, 10065. [2]West Virginia University Cancer Institute, West Virginia University, 1 Medical Center Drive, Morgantown WV, 26506 and [3]Wood Hudson Cancer Research Laboratory Inc, 931 Isabella Street, Newport Kentucky, 41701.

Running Title: NQO1 expression promotes NSCLC self-renewal and resistance

Key Words: NQO1, self-renewal, tumor-initiating cells, chemo-resistance, non-small cell lung cancer, proliferation, spheroids.

Abbreviations: NQO1, NSCLC

Corresponding Author: Erik A. Bey*

Competing interest statement: The authors have no outside competing interest regarding this article.

Author contributions: Erik A. Bey and Brian Madajewski contributed to the conception, experimental design, data acquisition, interpretation and analysis of data, manuscript preparation and submission of the article. Michael Boatman contributed to the experimental design, data acquisition and analysis for this article. Ivan Martinez and Julia Carter contributed to the manuscript preparation and submission of the article.

Supplemental Figure Legends

Supplemental Figure S 1. Spheroid culture does not affect cell viability. In **A**, the viability of cells in spheroid culture was evaluated via trypan blue exclusion over the two-week culture period. There was no obvious difference in cell viability between the A549-shCtr and A549-shNQO1 cell lines at any time over the course of observation. In **B**, cells in spheroid culture were evaluated for apoptosis induction by evaluating PARP1 cleavage, as well as AIF induction. A549 treated with 0.5 uM staurosporin [A549 (0.5 μM Stauro] served as a positive PARP1 cleavage positive control. There was no apparent induction of PARP1 cleavage, as well as no obvious increase in AIF expression over the spheroid culture period.

Supplemental Figure S 2. Loss of NQO1 via lentiviral shRNA inhibits spheroid formation, and spheroid formation is a result of clonal expansion. In **A**, Western bot analysis of lentiviral-shRNA knockdown of NQO1 [A549-shNQO1 (lenti)] in comparison to the A549 control cell line [A549-shNQO1 (lenti)] (left panel). In **B**, spheroid assay of A549-shCtr (lenti) and A549-shNQO1 (lenti) demonstrating a significant loss of spheroid formation with NQO1 reduction (** = p = 0.0066). In **C**, A549-sh-Ctr and A549-shNQO1 cells were tested for clonal spheroid forming ability in the presence of 1% methylcellulose, diluted 1:1 in 0.25% FBS/DMEM, as an alternative to 0.25% FBS/DMEM medium. Data show significant loss in spheroid forming ability in the A549-shNQO1 cell line in comparison to control, similar to what was observed in experiments with low serum and DMEM only (** = p = 0.0084).

Supplemental Figure S3. Inhibition of NQO1 activity results in loss of spheroid generating capabilities. In **A**, parental A549 cells were tested for their spheroid forming capabilities in both the presence and absence of the NQO1 inhibitor, dicoumarol. Cells were plated for the spheroid assay and treated with either vehicle control, or 50 μM dicoumarol and allowed to incubate for 2 weeks. Following incubation, the number of spheroids formed were counted. The inhibition of NQO1 lead to a significant decrease in the sphere forming abilities of the A549 cell line. In **B**, the same experiment was carried out using the H358 cell line, in which similar results were obtained.

Supplemental Figure S4. Spheroid cultured cells increase expression of stem cell markers, and are lost upon differentiation. A549-shCtr and A549-shNQO1 cells were pelleted prior to spheroid plating (shCtr and shNQO1), following two weeks of spheroid culture (shCtr 1° Sphere and shNQO1 1° Sphere), and following one week of differentiation in 10% FBS containing DMEM in an attached setting (shCtr 1° Sphere differentiated and shNQO1 1° Sphere differentiated). Western blot analysis was performed in order to visualize the expression of known stem cell markers SOX2, Shh, and Nanong. It was observed that there was an increase in the stem cell markers as a result of spheroid culture in both the A549-shCtr and A549-shNQO1 cell lines. The stem cell marker expression was subsequently decreased following plating in differentiating conditions.

Supplemental Figure S5. Spheroid culture conditions induce NQO1 expression in A549-shNQO1 cells. Both A549-shCtr and A549-shNQO1 cells grown in spheroid culture

for 2 weeks were assayed for NQO1 expression. In **A**, Western blot analysis demonstrating decreased expression of NQO1 in A549-shNQO1 spheres in comparison to A549-shCtr spheres. In **B**, real-time qRT-PCR analysis demonstrating a marked increase in NQO1 mRNA expression in spheroid cultured cells (A549-shNQO1) in comparison to A549-shNQO1 cells grown in attached conditions (A549-shNQO1 2D). (*** = p =0.0009) Additionally, it should be noted that the expression of NQO1 mRNA in the A549-shNQO1 sphere cultured cells remains significantly decreased in comparison to the A549-shCtr sphere cultured cells. (** = p =0.0018).

Supplemental Figure S6. Spheroid formation is dependent on NQO1 expression. In **A**, NQO1 expression was evaluated in the parental, shCtr, shNQO1, and 4C20 (second clone) H358 cell lines. The 4C20 clone expresses a greater amount of NQO1 than that of H358-shNQO1, and therefore was evaluated for its sphere forming abilities in **B**. In **B**, is the quantification of the spheroid formation assay, including our 4C20 cell line. It can be seen that with an increase in NQO1 expression there is an increase in spheroid formation, suggesting that a critical level of NQO1 expression is necessary to support spheroid formation.

Supplemental Figure S1

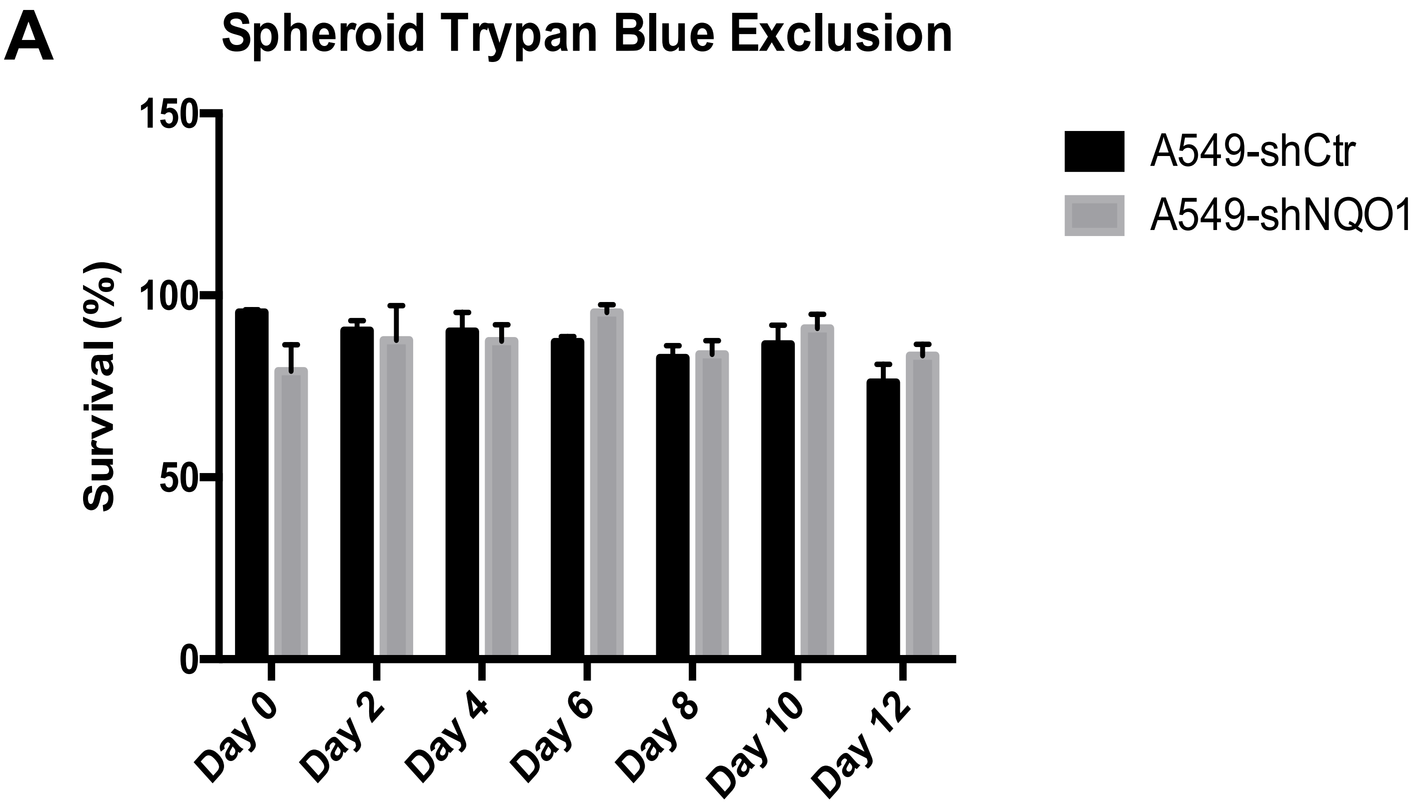

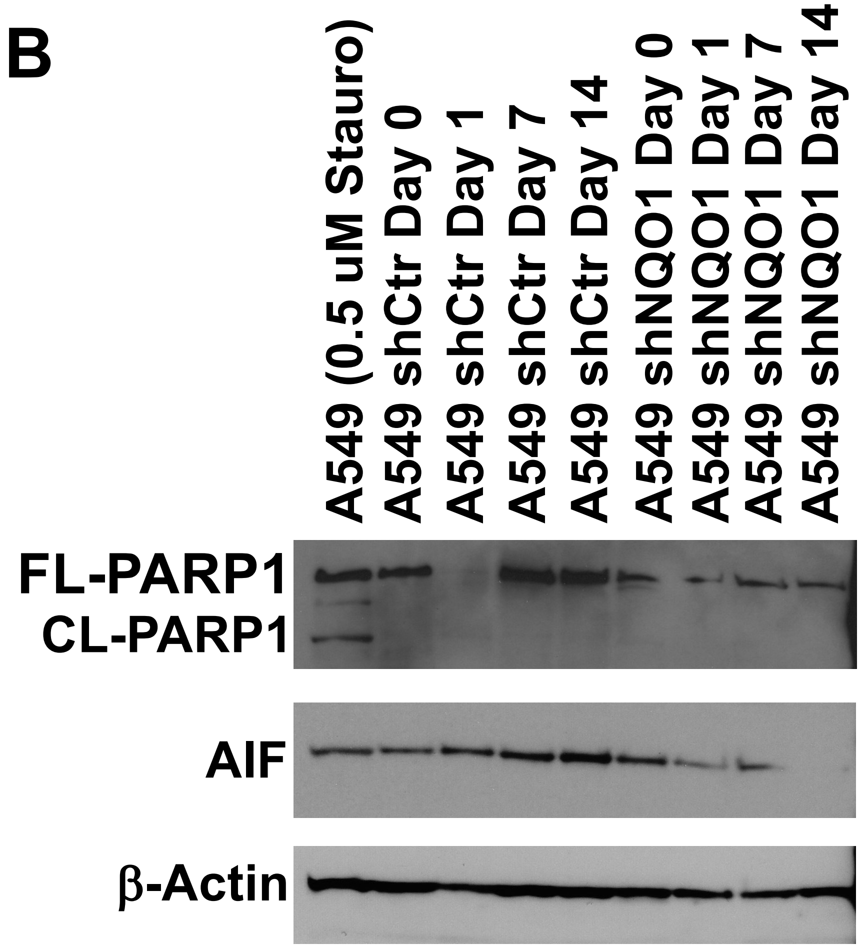

Supplemental Figure S2

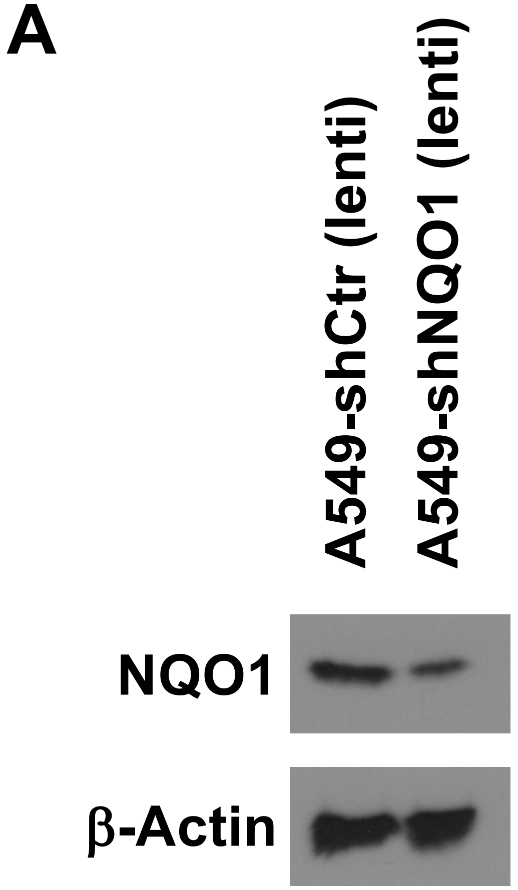

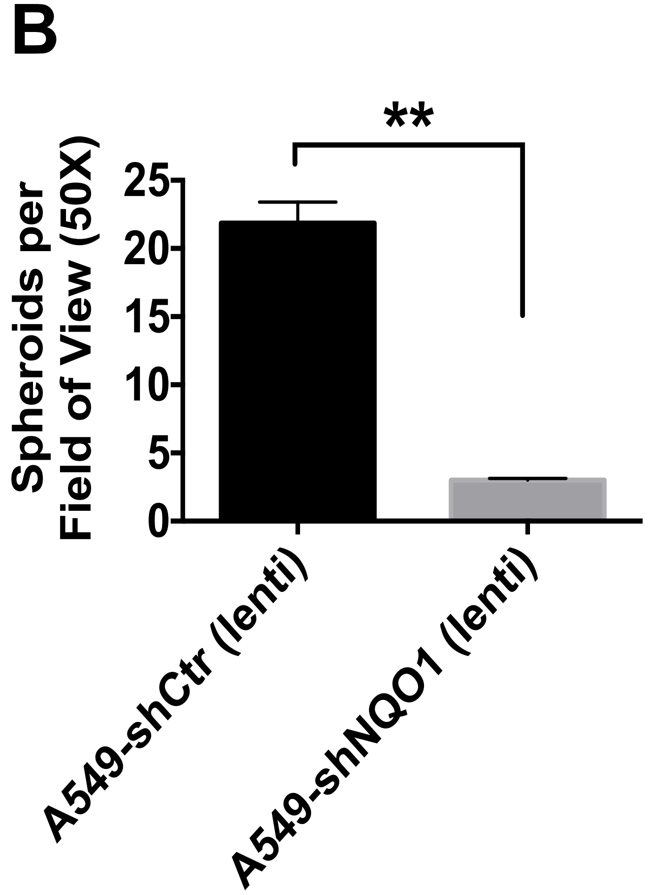

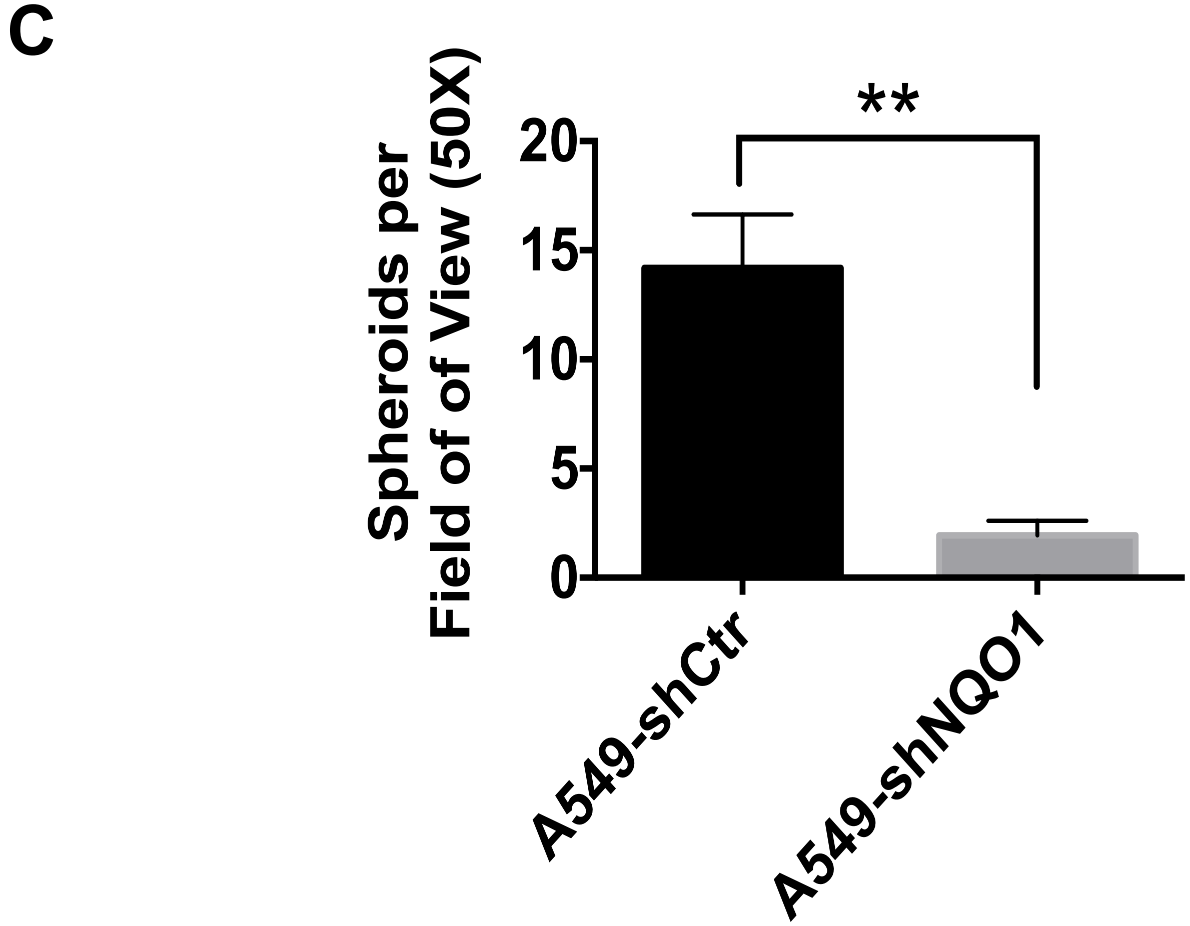

Supplemental Figure S3

A

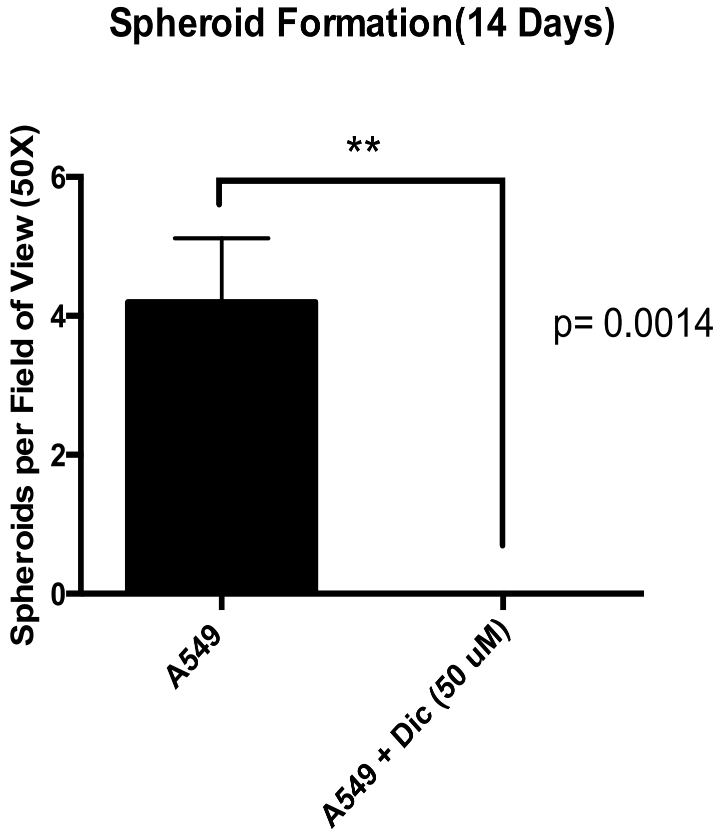

B

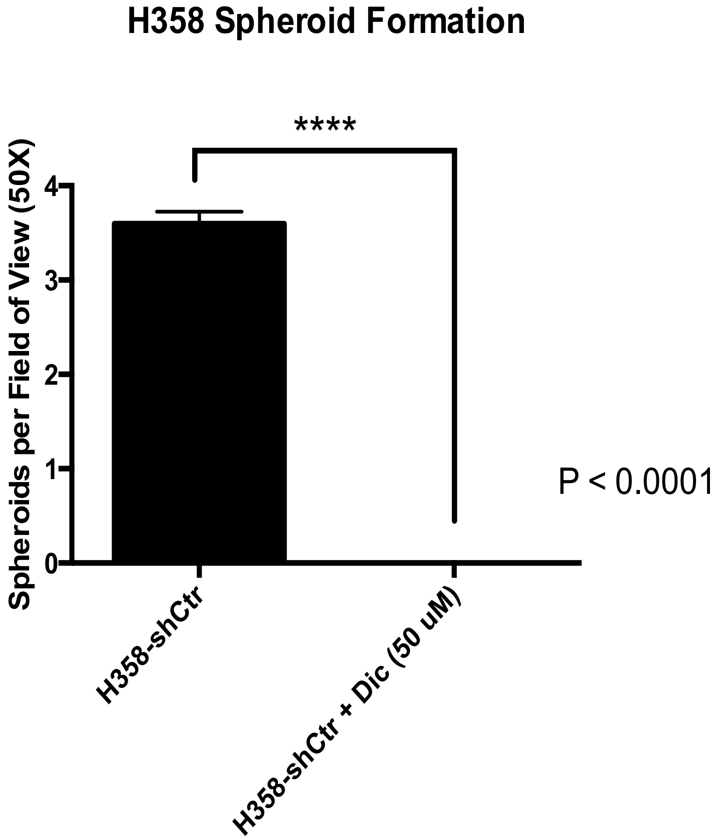

Supplemental Figure S4

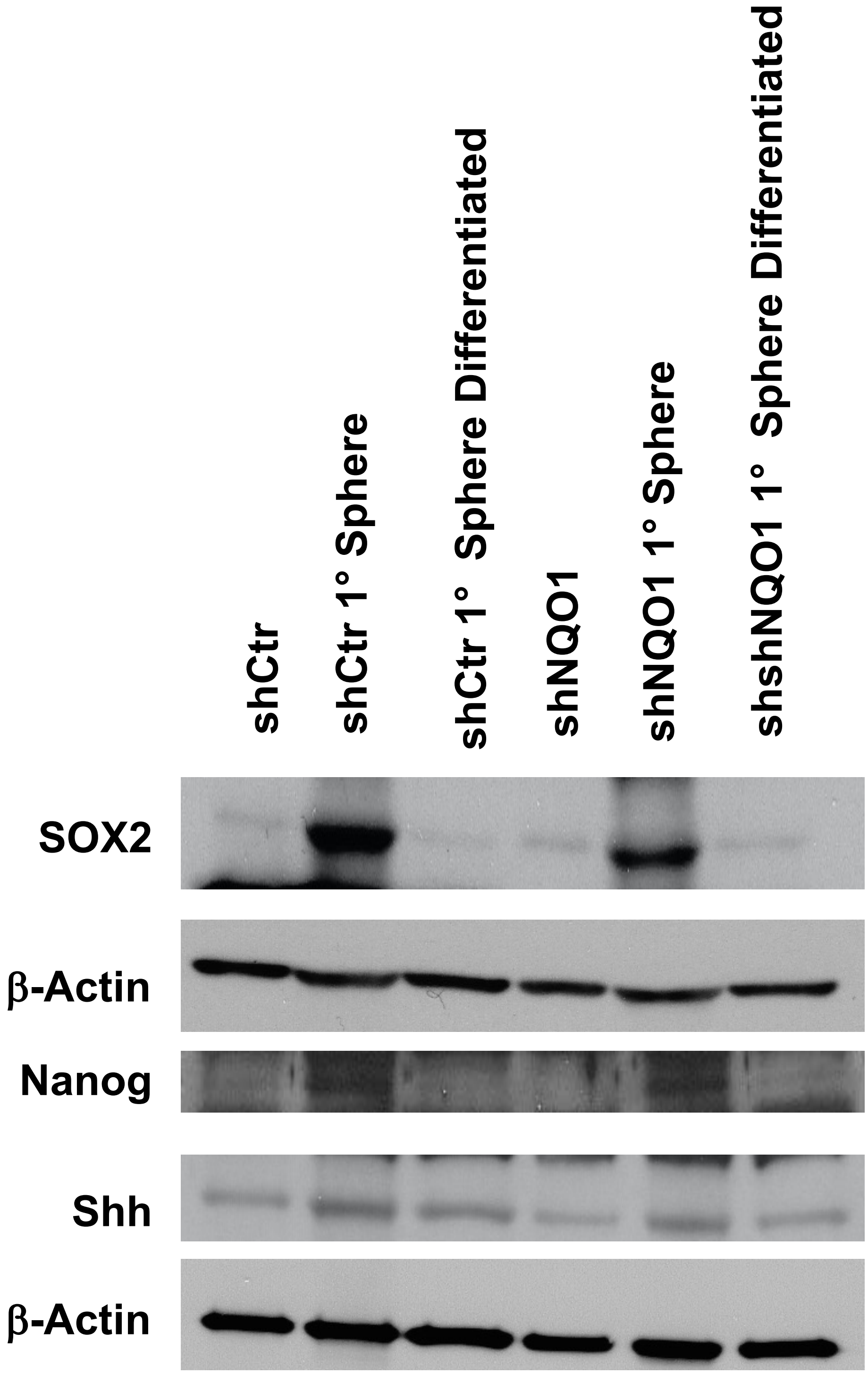

Supplemental Figure S5

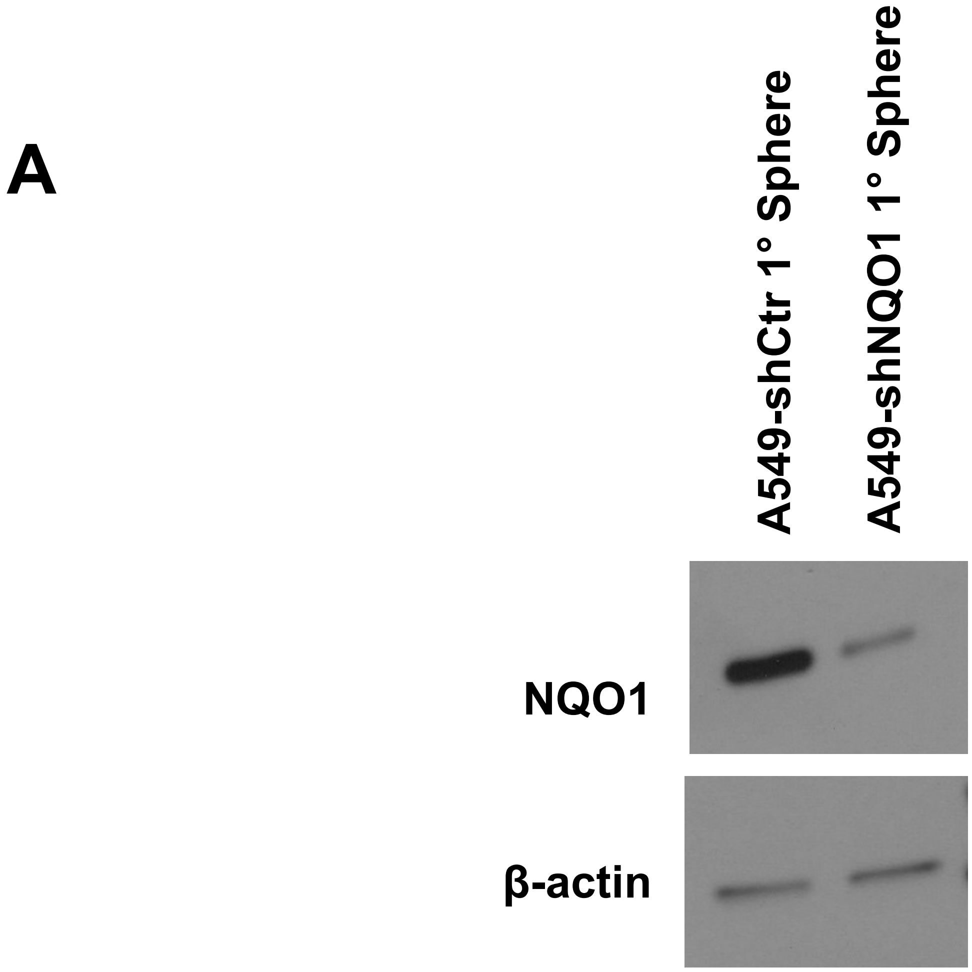

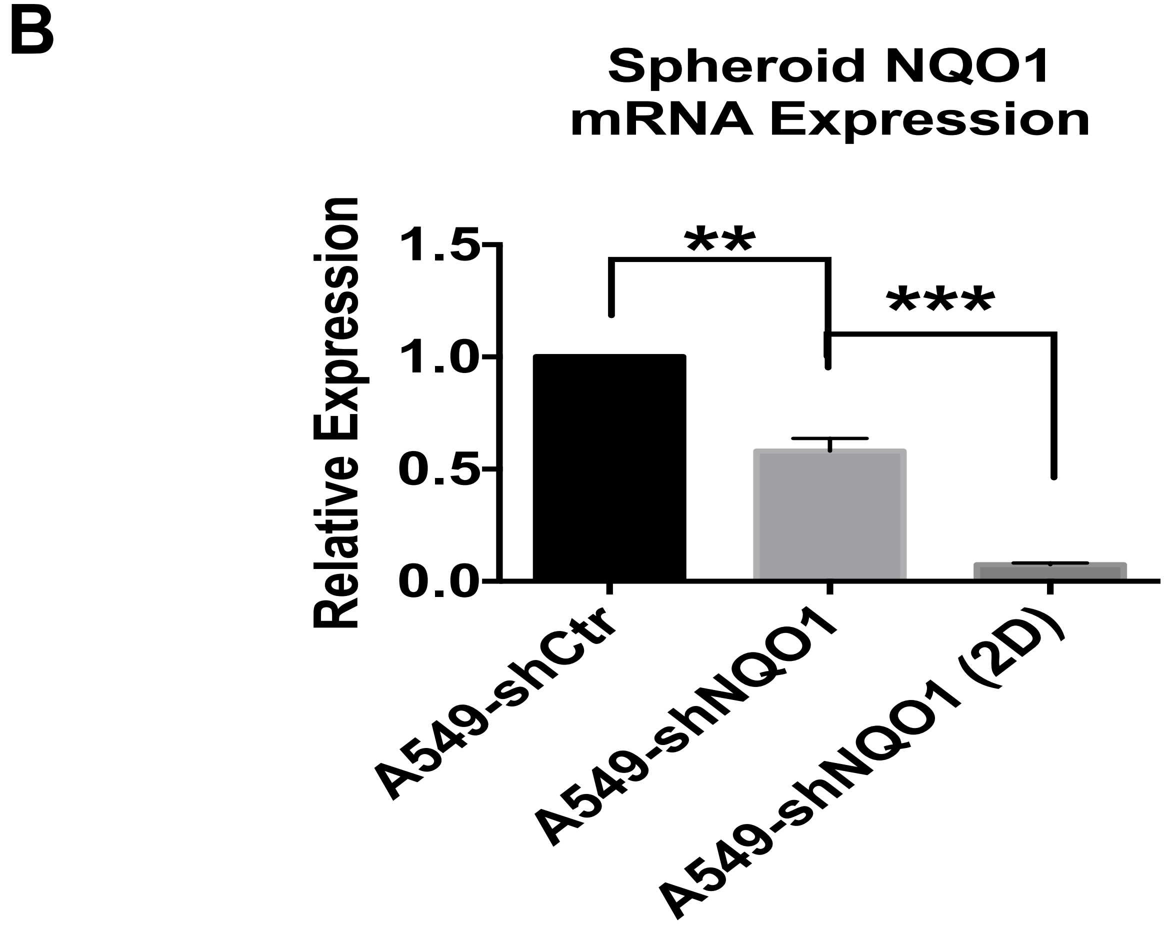

Supplemental Figure S6

A

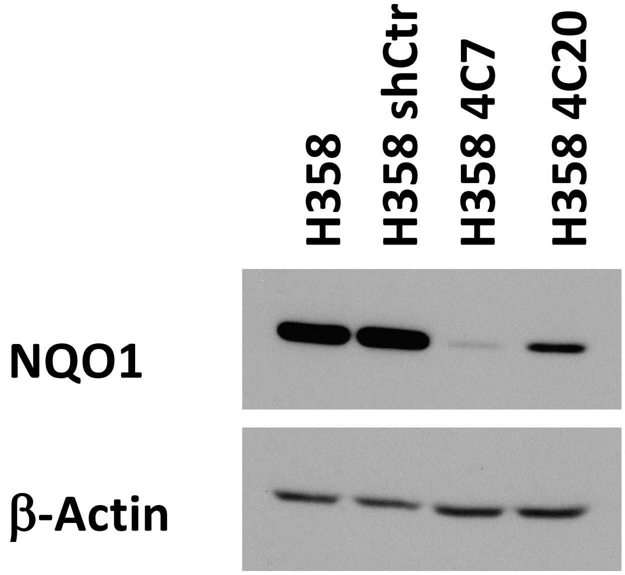

B

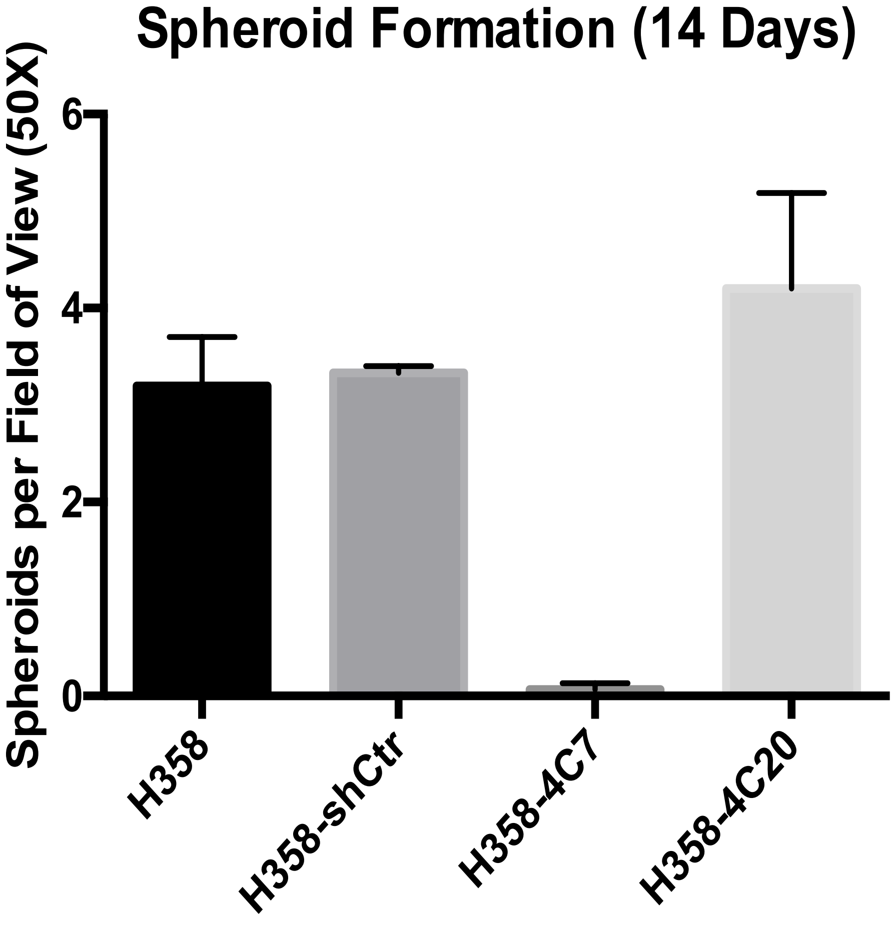

Supplement: Supplementary file 1 [file genes-14-00607-s001.zip › genes-2203852-supplementary.pdf]
